# Phenolic Profile and In Vitro Antioxidant Activity of Different Corn and Rice Varieties

**DOI:** 10.3390/plants12030448

**Published:** 2023-01-18

**Authors:** Corinne Bani, Chiara Di Lorenzo, Patrizia Restani, Francesca Mercogliano, Francesca Colombo

**Affiliations:** 1 Department of Pharmacological and Biomolecular Sciences, Università degli Studi di Milano, 20133 Milan, Italy; 2 Coordinating Research Center (CRC) “Innovation for Well-Being and Environment”, Università degli Studi di Milano, 20133 Milan, Italy

**Keywords:** pigmented cereals, phenolic compounds, in vitro antioxidant activity, celiac disease

## Abstract

Celiac disease (CD) is an autoimmune disease. To date, the only universally recognized treatment for CD is the gluten-free diet (GFD). Despite the GFD, a state of inflammation and oxidative stress could remain at the intestinal level of celiac patients. Several components of the diet, such as phenolic compounds with known antioxidant properties, could play a protective role in the inflammatory state of patients with CD. The objective of this study was the characterization of the phenolic profile and the antioxidant capacity of pigmented cereals (rice and corn) from the Italian market and farms. Different in vitro methods were applied: Folin–Ciocalteu assay, pH differential method, DPPH assay, TEAC assay, and High-Performance Thin Layer Chromatography technique. According to the results, pigmented varieties are possible valuable sources of phenolic compounds and anthocyanins with high antioxidant activity. They could be used as alternative ingredients for the formulation of gluten-free products.

## 1. Introduction

Celiac disease (CD) is an autoimmune pathology, characterized by a permanent intolerance to gluten, that occurs in genetically predisposed individuals. If untreated, this pathology can progress, leading to a complete atrophy of the small intestine that determines the malabsorption of nutrients [1]. CD affects about 1.4% of the world’s entire population, registering, in recent years, an increase in incidence and prevalence driven by diagnostic improvement [2,3].

To date, the only effective treatment for individuals with CD is a permanent gluten-free diet (GFD). This dietetic therapy allows the solution of typical symptoms of CD (e.g., chronic diarrhea, bloating, weight loss, intestinal malabsorption, nausea, and vomiting), reducing the risk of occurrence of associated complications [4,5,6]. 

From studies in the literature, it appears that, for most patients with CD, a suitable GFD determines a remission of both intestinal and extra-intestinal symptoms, with subsequent restoration of the intestinal mucosa [7,8,9]. There is also a normalization of serum levels of gluten-dependent antibodies such as anti-gliadin, anti-deamidated gliadin peptides, anti-endomysium, and anti-transglutaminase antibodies [5]. 

Corn and rice are among the most frequently consumed cereals in GFD; their pigmented varieties could be used as innovative ingredients to improve the quality of the celiac diet [10].

Pigmented cereals, in fact, have better sensory characteristics and health properties compared with usual varieties. Several in vitro and cellular studies have demonstrated their potential beneficial effects, including antioxidant, probiotic, hypoglycemic, and hypolipidemic effects, which depend on the high content of bioactive compounds [11,12,13,14,15,16,17]. 

The most interesting nutrients are present in the outermost part of the cereals’ caryopsis: this portion is rich in fibers, minerals, vitamins, and bioactive compounds such as phenolic compounds. 

Some bioactive compounds have antioxidant properties that can contribute to the neutralization of radical species, interrupting their formation and, in parallel, stimulating the body’s endogenous antioxidant defenses. Phenolic compounds are secondary metabolites of plants; some are essential for plant development while others are produced in response to stress conditions such as infections and ultraviolet radiation [18,19]. 

These compounds are present in the plants in soluble and insoluble/bonded forms to cell wall components. In corn, phenolic compounds are mainly present in the bound form [20] while, according to the literature, pigmented rice varieties are particularly rich in free phenolic compounds [21]. The soluble fraction represents a good source of dietary antioxidants [22].

Corn and rice are particularly rich in ferulic acid and p-coumaric acid. In corn, ferulic acid (or 3-methoxy-4-hydroxycinnamic acid) is the most abundant compound, and it is mainly located in the aleurone and pericarp area; when oxidized, it tends to form dimers and trimers. Flavonoids present in corn and rice include flavonols (e.g., quercetin and kaempferol) [23,24], while pigmentated varieties are particularly rich in anthocyanins, a class of water-soluble molecules responsible for their color [25]. 

Several preliminary studies conducted in vitro and in vivo have shown their potential role in reducing some risk factors for cardiovascular disease, diabetes, obesity, cancer, and chronic diseases [15].

The aims of the present study were the following: (1) the collection of cereal samples (rice and corn) with different pigmentations from the Italian market and Italian farms. In particular, rice samples were obtained from the Italian market and “Azienda Agricola Bertolone Eleonora di Bertolone Giovanni”—Collobiano (VC); corn samples were supplied by the Italian farm “Associazione Rosso Mais”—Rovetta (BG); “Azienda Agricola Caretto”—San Giorgio C.se (TO). In addition, six varieties of *Zea mays* L. were provided by the “Bioscience Department—Università degli Studi di Milano.” (2) The characterization of the phenolic content and antioxidant capacity of the selected samples.

## 2. Results and Discussion

### 2.1. Total Anthocyanin Content

Anthocyanins provide different colors to the grain, from red to purple, and have beneficial effects on human health [26].

However, some pigmented varieties do not contain anthocyanins, so the color could be due to proanthocyanidins [20,27].

Table 1 shows the total anthocyanin content of pigmented rice and corn varieties, expressed as cyanidin-3-O-glucoside equivalents (mg CY/g). The results of the samples were statistically compared to underline similarities or differences between sample varieties.

According to previous studies, the total anthocyanin content decreases in rice varieties as follows: purple > black > red > brown [27]. Violet brown rice (VIO) showed the highest anthocyanin content (3.98 mg CY/g) when compared to Nerone (NRN 3.04 mg CY/g) and Venere (VEN 1.18 mg CY/g).

The quantitative data obtained in this study agree with those published by Hosoda and co-workers, where the total anthocyanin content in black varieties ranged between 0.70 and 5.05 mg CY/g [28].

The total anthocyanin content was measurable in the corn pigmented varieties Pop BRP, Su/Su BRP, and Scagliolo BRP, and the results agreed with previously published data (0.015–8.60 mg CY/g [29] and 0.014–0.74 mg CY/g [26]). The Su/Su BRP corn had the highest anthocyanin content (1.05 mg CY/g) when compared to Scagliolo BRP (0.52 mg CY/g) and Pop BRP (0.45 mg CY/g).

### 2.2. Soluble Polyphenols Content

Phenolic acids are the most abundant polyphenols in grain, although other compounds, such as flavonoids (among which are anthocyanins), have been described [30]. Pigmented varieties contain different bioactive compounds, including anthocyanins, proanthocyanidins, carotenoids, and phenolic acids [26]. 

This study focused on the soluble fraction, since free phenolic compounds are rapidly absorbed in the small intestine, an anatomical site involved in several chronic diseases (celiac disease, among others) [31,32].

The pigmented cereal varieties, rich in anthocyanins, could be interesting gluten-free ingredients. 

Different samples of pigmented varieties were included in this research to investigate their suitability for celiac patients. In parallel, some non-pigmented varieties were used for comparison.

#### 2.2.1. Rice

The soluble phenolic content (SPC) of rice samples, determined by the Folin–Ciocalteu’s assay, is reported in Table 2. The results of the samples were statistically compared to underline similarities or differences between varieties of samples.

The SPC for rice samples ranged between 0.72 and 7.39 mg GAE/g of grain flour. The SPC of pigmented varieties was significantly higher than that measured in the white sample Originario brown (OR). As expected, brown and black varieties showed the highest SPC, which was higher for Nerone brown (NRN) than for Violet brown (VIO) and Venere brown (VEN). These values agree with data from the literature; Mellini and co-workers reported a soluble polyphenol content (SPC) in pigmented rice samples ranging between 5.44 ± 0.14 and 15.08 ± 0.29 mg GAE/g dry matter basis [21]. Yodmanee and co-workers detected a soluble polyphenol content (SPC) in eight pigmented rice samples ranging between 0.059 ± 0.07 and 3.29 ± 0.07 mg GAE/g dry matter basis [33]. 

#### 2.2.2. Corn

The soluble phenolic content (SPC) of corn samples, determined by the Folin–Ciocalteu assay, is reported in Table 3. The results of the samples were statistically compared to underline similarities or differences between the varieties.

Corn varieties showed a variable content of total soluble polyphenol compounds (SPC), ranging between 1.04–4.27 mg GAE/g (Table 3). Data on soluble polyphenol content (SPC) in corn varieties agree with the results of Lopez-Martinez and co-workers, showing that different pigmented phenotypes, from white to purple, have a SPC ranging between 0.33 and 6.80 mg GAE/g [29].

Generally speaking, the non-pigmented varieties showed the lowest SPC, apart from some samples, such as Ottofile brown 2019 (OTb) and Nostrano Isola brown 2018 (NSb-2018). The varieties characterized by the presence of anthocyanins (Pop BRP, Su/Su BRP and Scagliolo BRP) (Table 1) also contained the highest content of soluble phenolic compounds. However, although the Rosso di Rovetta brown (RRb) sample did not contain anthocyanins, it presented an interesting level of phenolic compounds (2.17 ± 0.02 mg GAE/g). 

### 2.3. Total Antioxidant Capacity

To evaluate the in vitro antioxidant activity of the samples, two different spectrophotometric methods were applied: DPPH assay and TEAC assay. 

DPPH assay is an excellent tool for monitoring the chemical reactions involving radicals. DPPH is a stable free radical with a characteristic absorption at 517 nm, which decreases significantly when it reacts with proton radical scavengers [34]. 

The Trolox Equivalent Antioxidant Capacity (TEAC) method is based on the measure of the loss of color when an antioxidant is added to the blue-green chromophore ABTS+• (2,2-azino-bis(3-ethylbenz- thiazoline-6-sulfonic acid)). The antioxidant compounds reduce ABTS+• to ABTS and decolorize it [34]. 

The in vitro antioxidant activity (AOA) of rice and corn samples, determined by the DPPH assay and by the TEAC assay, is reported in Table 4. The results of the samples were statistically compared to underline similarities or differences between varieties of samples.

For rice varieties, the in vitro antioxidant activity evaluated by the DPPH assay ranged between 1.39 and 2.26 mg GAE/g for black varieties, between 0.89 and 1.70 mg GAE/g for red varieties, and was 0.14 mg GAE/g for Originario (OR) sample. 

For rice varieties, the in vitro antioxidant activity evaluated by the TEAC assay ranged between 14.25 and 8.22 mg TE/g for black varieties, from 9.20 and 5.28 mg TE/g for red varieties, and was 1.18 mg TE/g for Originario (OR) sample.

The in vitro antioxidant activity evaluated by both spectrophotometric assays showed that the pigmented varieties of rice had higher AOA compared to the non-pigmented one. 

The in vitro antioxidant activity evaluated of corn varieties by DPPH assay ranged between 0.736 and 0.138 mg GAE/g for red and red/violet varieties, between 0.197 and 0.091 mg GAE/g for yellow ones, and was 0.098 mg GAE/g for Ostenga (OSb) sample.

The in vitro antioxidant activity evaluated of corn varieties by TEAC assay ranged between 10.58 and 2.19 mg TE/g for red and red/violet varieties, between 2.80 and 1.81 mg TE/g for yellow ones, and was 1.83 mg TE/g for Ostenga (OSb) sample.

Among the corn samples, the anthocyanin-rich varieties, and the Rosso di Rovetta brown (RRb) had the highest antioxidant capacity.

In order to evaluate the contribution of phenolic compounds to the in vitro antioxidant activity of the samples, different variables were compared.

**Figure 1:**
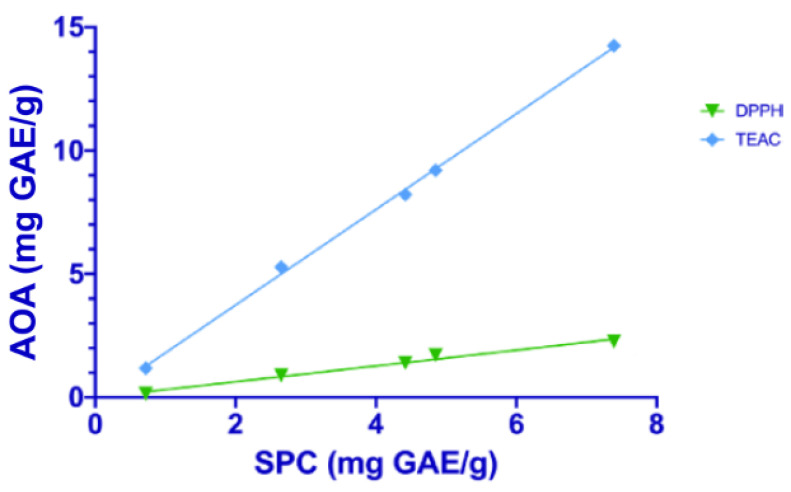
Linear regression between soluble phenolic content (SPC) and the in vitro antioxidant activity (AOA), measured with DPPH (green line) and TEAC assays (blue line) in rice samples.

**Figure 2:**
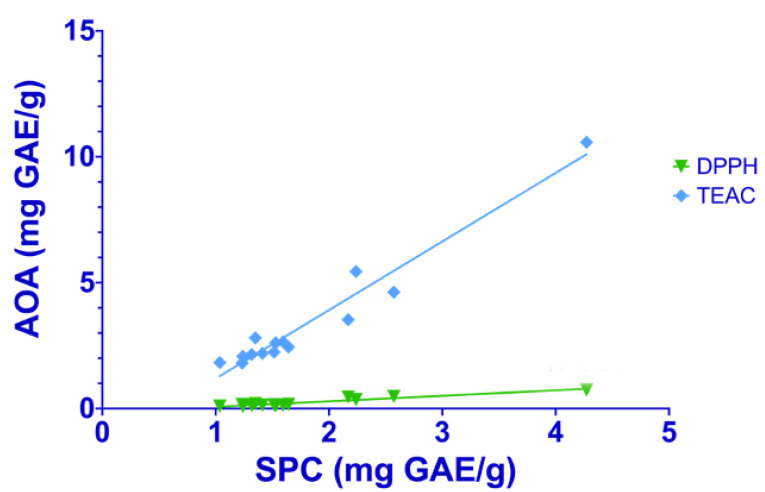
Linear regression between soluble phenolic content (SPC) and the in vitro antioxidant activity (AOA) measured with DPPH (green line) and TEAC assays (blue line) in corn samples.

Data on in vitro antioxidant activity for each sample, measured with the different spectrophotometric assays, were correlated to soluble polyphenol content (Figure 1 and Figure 2).

A positive linear correlation was found in rice samples between total polyphenol content and in vitro antioxidant activity evaluated with the DPPH (r = 0.989, *p* < 0.01) and TEAC (r = 0.995, *p* < 0.01) assays. Even for corn samples, a similar correlation was found with the DPPH (r = 0.947, *p* < 0.01) and TEAC (r = 0.955, *p* < 0.01) assays. Therefore, the phenolic compounds are directly associated with antioxidant activity.

### 2.4. High Performance Thin Layer Chromatography

The phenolic profiles of the samples were characterized using the HPTLC technique. In addition, to obtain a semi-quantitative evaluation of the in vitro antioxidant capacity (AOA) of samples, the DPPH was selected as the derivatization agent and the plate was exposed at 254 nm, 366 nm, and visible light.

The variation in color, from violet to yellow, was proportional to the AOA of each compound contained in the samples.

#### 2.4.1. Validation Data

The *Ratio frontis* (Rf) and the Limit of Detection (LOD) of each analyzed standard are reported in Table 5. The LODs were determined by loading onto the plate and decreasing the concentration of standard solutions.

#### 2.4.2. Rice

**Figure 3:**
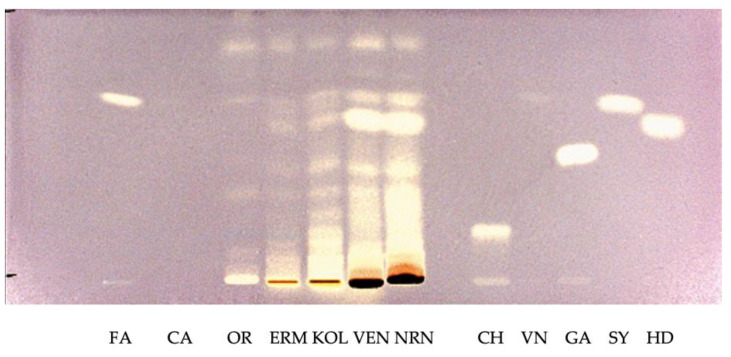
HPTLC patterns of the composition of phenolic compounds of the ethanol fraction of rice varieties detected at visible light after derivatization with DPPH solution. Standard phenolic acids are run in parallel. FA: ferulic acid, CA: coumaric acid, CH: chlorogenic acid, VN: vanillic acid, GA: gallic acid, SY: syringic acid, HD: dihydroxybenzoic acid, OR: Originario brown, ERM: Ermes brown, KOL: Kolorado brown, VEN: Venere brown, NRN: Nerone brown.

Figure 3 shows the HPTLC profile of the composition of phenolic compounds of the ethanol fraction of rice samples, detected at visible light after derivatization with DPPH solution. 

The in vitro antioxidant activity of coumaric acid (CA) and vanillic acid (VN) was not detectable. Although the complex fingerprints of the samples decrease the possibility of identifying all compounds separated during the chromatographic run, bands with the same *Ratio frontis* (Rf) of ferulic acid (Rf = 0.60) were visible in all rice samples at visible light. In the pigmented varieties, a band that could be attributed to dihydroxybenzoic acid (Rf = 0.54) was visible. Although the HPTLC method, due to the dark color of these molecules, does not completely take into account the contribution of anthocyanins to the AOA, it is interesting to note that the black rice varieties, Venere brown (VEN) and Nerone brown (NRN), as well as the red sample Kolorado brown (KOL), showed the highest content of antioxidant compounds compared to other samples. The HPTLC data agree with the data obtained from the spectrophotometric analysis.

#### 2.4.3. Corn

**Figure 4:**
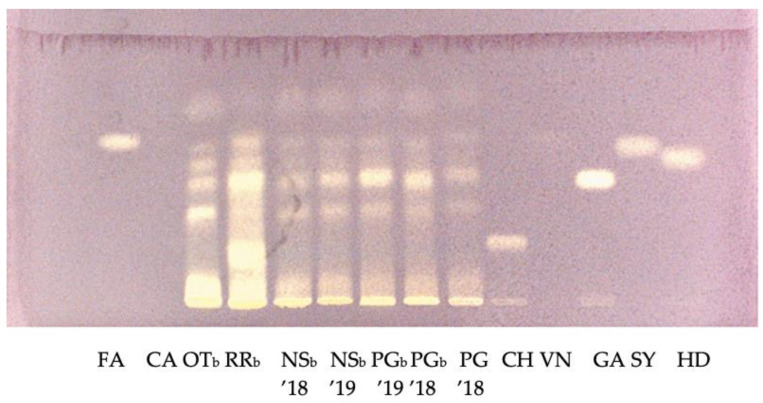
HPTLC patterns of the composition of phenolic compounds of the ethanol fraction of corn varieties detected at visible light after derivatization with DPPH solution. Standard phenolic acids are run in parallel. FA: ferulic acid, CA: coumaric acid, CH: chlorogenic acid, VN: vanillic acid, GA: gallic acid, SY: syringic acid, HD: dihydroxybenzoic acid, OTb: Ottofile brown 2019, RRb: Rosso di Rovetta brown, NSb ’18: Nostrano Isola brown 2018, NSb ’19: Nostrano Isola brown 2019, PGb ’19: Pignoletto brown 2019, PGb ’18: Pignoletto brown 2018, PG ’18: Pignoletto 2018.

**Figure 5:**
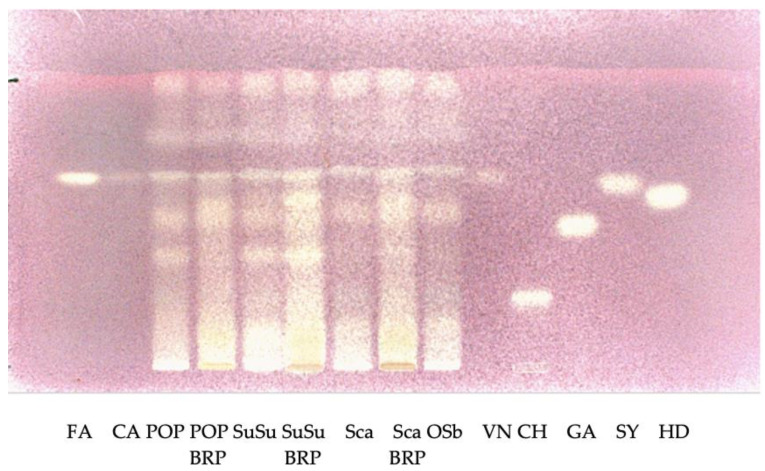
HPTLC patterns of the composition of phenolic compounds of the ethanol fraction of corn varieties detected at visible light after derivatization with DPPH solution. Standard phenolic acids are run in parallel. FA: ferulic acid, CA: coumaric acid, CH: chlorogenic acid, VN: vanillic acid, GA: gallic acid, SY: syringic acid, HD: dihydroxybenzoic acid, Sca: Scagliolo, ScaBRP: Scagliolo BRP, OSb: Ostenga brown.

Figure 4 and Figure 5 show the HPTLC patterns of the composition of phenolic compounds of the ethanol fraction of corn samples detected at visible light after derivatization with DPPH solution.

The corn samples showed complex fingerprints. The pigmented corn varieties Pop BRP, Su/su BRP, Scagliolo BRP, and Rosso di Rovetta brown (RRb) showed the highest content of antioxidant compounds. Interestingly, the yellow variety Ottofile brown 2019 (OTb) showed a high in vitro antioxidant capacity compared to the other non-pigmented samples.

Phenolic acids, in particular ferulic and p-coumaric, are the main phenolic compounds in corn [35]. Bands with the same Rf of ferulic acid (Rf = 0.64) were visible in all pigmented and non-pigmented samples. In addition, for corn samples, the HPTLC results agreed with the data obtained from spectrophotometric analysis.

## 3. Materials and Methods

### 3.1. Samples

**Figure 6:**
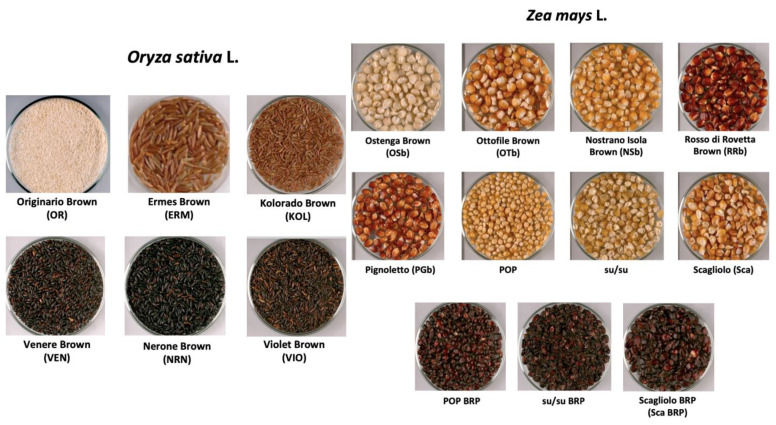
Rice and corn samples included in this study.

Rice and corn samples with different pigmentations were included in the study (Table 6). Rice samples were from the Italian market and “Azienda Agricola Bertolone Eleonora di Bertolone Giovanni’’—Collobiano (VC), while corn samples were provided by the Italian farm “Associazione Rosso Mais”—Rovetta (BG); “Azienda Agricola Caretto”—San Giorgio C.se (TO) (Figure 6).

In addition, six varieties of *Zea mays* L. were provided by the “Bioscience Department—Università degli Studi di Milano” and these included three yellow varieties (Pop, Su/su, and Scagliolo) and three pigmented varieties (Pop BRP, Su/su BRP, and Scagliolo BRP), obtained through recurrent backcrosses between the above reported yellow varieties and the synthetic purple corn variety carrying B1 and Pl1 alleles (used for animal feeding) [36]. A portion of each sample was ground using an electric mill (Novital, Italy) to obtain the corresponding cereal flours.

### 3.2. Determination of Total Anthocyanin Content

The total anthocyanin content was determined by spectrophotometric analysis in pigmented corn and rice samples in accordance with the AOAC method, based on the pH differential assay [37].

#### 3.2.1. Extraction of Anthocyanins

First, 0.5 g of ground whole grain was extracted with 10 mL of methanol:1 M HCl 85:15 (*v/v*) and maintained under stirring in the dark for 30 min. Samples were centrifuged at 8000 g for 20 min at 4 °C (Avanti J-25, Beckman Coulter, CA, USA), filtered with a 0.45 μm PTFE filter (VWR International, Fontenay-sous-Boys, Francia), and stored at −20 °C until used. Each extraction was performed in triplicate.

#### 3.2.2. pH Differential Assay

The absorbance of the samples, suitably diluted with pH 1.0 (0.025 M potassium chloride) and pH 4.5 (0.4 M sodium acetate) buffers, was measured both at 520 and 700 nm, correcting for haze using the last reading. Each analysis was performed in triplicate. 

Total anthocyanin pigments (AP) are expressed as cyanidin-3-O-glucoside equivalents (CY mg/g), according to the following Equation (1):

AP (CY mg/g) = ΔA × MW × DF × 1000 × V/e × l × W(1)

where: ΔA is the difference between (A_520 nm_ − A_700 nm_) at pH 1.0 and (A_520 nm_ − A_700 nm_) at pH 4.5; MW is the molecular weight (449.2 g/mol for cyanidin-3-O-glucoside); DF is the dilution factor; 1000 is the factor for conversion from g to mg; V is the extraction volume (L); e is the molar extinction coefficient (26,900 for cyanidin-3-O-glucoside); l is the path length in cm (1 cm); and W is the sample weight (g).

### 3.3. Extraction of Soluble Phenolic Compounds

Soluble phenolic compounds were extracted as follows: samples of 0.5 g of ground whole grain were added with 10 mL of ethanol:water 60:40 (*v/v*) and maintained under stirring in the dark for 2 h. Samples were centrifuged at 2000 g for 15 min at 4 °C (Avanti J-25, Beckman Coulter, CA, USA). The extracts were filtered with 0.45 μm filters (VWR International, Fontenay-sous-Boys, Francia) and stored at −20 °C until used. Each extraction was performed in triplicate. These extracts were used to determine soluble phenolic content and in vitro antioxidant activity, as well as for the separation of different classes of molecules by High Performance Thin Layered Chromatography.

### 3.4. Determination of Soluble Phenolic Content

Phenolic compounds can be found in cereals bound to cell wall components or in soluble form. This study is focused on the soluble fraction, since it allows the differentiation of the pigmented varieties from the non-pigmented ones. The soluble phenolic compounds were obtained by extracting samples with a hydroalcoholic solution.

#### Folin–Ciocalteu’s Assay

The Folin-Ciocalteu method, as reported by Singleton et al. [38], was used to determine the soluble polyphenol content.

Aliquots of 300 μL from different suitably diluted samples (or water for blank) were mixed in test tubes with 1.5 mL of 0.2 N Folin–Ciocalteu’s reagent, and 1.2 mL of 7.5% sodium carbonate. After 30 min in the dark, the absorbance was measured at 765 nm by UV-visible spectrophotometer analysis (Varian Cary 50 SCAN, Palo Alto, CA, USA). Results were expressed as equivalents of gallic acid (GAE) in mg/g. The polyphenols contained in the samples were calculated using a standard curve of gallic acid ranging from 5 to 50 mg/mL. Each analysis was performed in triplicate.

### 3.5. Determination of In Vitro Antioxidant Capacity

#### 3.5.1. DPPH Assay

The in vitro antioxidant activity (AOA) of samples was evaluated by spectrophotometric analysis as a measure of radical scavenging activity using 1,1-diphenyl-2-picryl-hydrazyl free radical (DPPH) [39]. Aliquots of 1 mL of 0.005% DPPH in methanol were mixed with 0.5 mL of each prepared sample and suitably diluted. The absorbance was measured at 517 nm after 30 min. The concentration of antioxidants was calculated using a calibration curve of gallic acid, ranging from 1.0 to 5.0 μg/mL, and expressed as equivalents of gallic acid (GAE) in mg/g.

#### 3.5.2. TEAC Assay

The Trolox Equivalent Antioxidant Capacity (TEAC) assay was performed as described by Re et al. [40], with some modifications. 

An ABTS radical cation solution was prepared by mixing 7 mM ABTS (1:1 *v/v*) and 2.45 mM of potassium persulfate; the mixture was then maintained in the dark at room temperature for 12–16 h. Before use, the ABTS+• solution was diluted with ethanol to reach an absorbance of 0.7 ± 0.02 at 734 nm. Aliquots of 1.5 mL of ABTS+• solution were mixed with 150 μL of each sample (or water as a blank), prepared and suitably diluted. The absorbance was measured at 734 nm after 6 min. 

The percentage of inhibition of ABTS+• was calculated with the following Equation (2):

% Inhibition of ABTS+• = [(Ab − At)/Ab] × 100,(2)

where Ab is the absorbance of the blank and At is the absorbance of the test. 

Results were calculated using a standard curve of Tolox, ranging from 10 to 30 μg/mL, and expressed as mg/g of Trolox equivalents (mg TE/g).

#### 3.5.3. High Performance Thin Layer Chromatography 

High Performance Thin Layer Chromatography (HPTLC) is a fast and suitable method for the screening of different classes of molecules, allowing for the fingerprint characterization of complex products [41]. Moreover, the HPTLC technique allows for the evaluation of some biological properties, which can be directly associated with any specific compound. Among the possible applications, the semi-quantitative measure of antioxidant activity is described herein. 

Particularly, in this study, the HPTLC technique was applied to evaluate the in vitro antioxidant activity associated with cereals’ flavonoids, separated by chromatography.

Soluble phenolic compounds were extracted as previously described for spectrophotometric analysis; after centrifugation, the supernatants were dried through N_2_ flow and solubilized in 0.5 mL of methanol.

Five μL of phenolic acids (coumaric acid, ferulic acid, chlorogenic acid, vanillic acid, gallic acid, syringic acid, and dihydroxybenzoic acid) standard solutions (200 μg/mL) were applied on silica-gel plates, 254F (10 × 20 cm, Merck, Darmstadt, Germany), using a semi-automatic applicator, Linomat 5 (Camag, Muttenz, Svizzera). For the cereal samples, volumes of 15 μL were loaded onto the plate. The chromatographic run was performed by using a mobile phase (10 mL) containing acetone:toluene:formic acid 4.5:4.5:1 (*v/v/v*).

After the chromatographic run, the plate was exposed at 254 nm, derivatized with a DPPH methanolic solution (0.05%), and dried in an extractor hood at room temperature for 1 min. The dried plate was wrapped with aluminum foil for 30 min and then exposed to a UV lamp (254 and 366 nm) and to visible light. The images were captured by using a specific software called VisionCats (CAMAG, Switzerland).

### 3.6. Statistical Analysis

Data were subjected to one-way analysis of variance (ANOVA), followed by the Duncan test, to evaluate statistical differences between cereal samples (threshold for statistical significance: *p* < 0.05). To evaluates the correlation between variables (threshold for statistical significance: *p* < 0.01), the Pearson test was applied. Statistical analyses were carried out with IBM SPSS Statistics, Version 27.0.

## 4. Conclusions

This study focused on rice and corn varieties, with particular attention paid to the pigmented varieties. Some of the collected rice and corn variants were available on the Italian market and farms. The pigmented grain varieties represent a good source of useful bioactive compounds in the diet of the general population but could be particularly important for patients with CD.

Considering the oxidative imbalance and chronic inflammation in celiac subjects, which are only partially solved by the gluten-free diet, the identification and selection of novel ingredients characterized by antioxidant properties could provide numerous benefits. 

Pigmented cereals are excellent candidates for gluten-free products because they are rich in phenolic compounds (among which are anthocyanins) and molecules with high antioxidant activity, and they are naturally gluten-free. This is the first study that has evaluated the possible role of gluten-free pigmented cereals in modulating oxidative stress in celiac patients. The in vitro methods applied in this study allowed for the characterization of different corn and rice samples, evaluating their antioxidant capacity in parallel. 

In general, pigmented cereals have an interesting phenolic profile, particularly the anthocyanin-rich varieties. The polyphenol content was found to be proportional to the in vitro antioxidant activity assessed by the different spectrophotometric (DPPH and TEAC tests) and chromatographic (HPTLC) methods.

The results obtained in this study could be useful to select naturally gluten-free ingredients, rich in polyphenols. These ingredients could enhance the nutritional/sensorial quality of gluten-free foods, improving the quality of life of patients with celiac disease.

## Figures and Tables

**Table 1:** Total anthocyanin content (TA) of pigmented rice and corn varieties.

Cereals	Sample Code	Kernel Colour	TA (mg CY/g) Mean ± SD
Rice	ERM	Red	ND
	KOL		ND
	VEN	Black	1.18 ± 0.07 ^a^
	NRN		3.04 ± 0.18 ^b^
	VIO		3.98 ± 0.38 ^c^
Corn	RRb	Red	ND
	PG_2018		ND
	PGb_2018		ND
	PGb_2019		ND
	Pop BRP	Red/Violet	0.45 ± 0.07 ^a^
	Su/Su BRP		1.05 ± 0.06 ^b^
	Scagliolo BRP		0.52 ± 0.06 ^a^

Data are expressed as cyanidin-3-O-glucoside equivalents (mg CY/g) (*n* = 3); ND: <LOD: 0.4 mg/g. Data with different letters in the same column for same cereal are significantly different (*p* < 0.01).

**Table 2:** Soluble phenolic content (SPC) of rice varieties.

Sample Code	Kernel Colour	SPC (mg GAE/g) Mean ± SD
OR	White	0.72 ± 0.06 ^a^
ERM	Red	2.65 ± 0.16 ^b^
KOL		4.84 ± 0.21 ^c^
VEN	Black	4.42 ± 0.36 ^c^
NRN		7.39 ± 0.22 ^d^
VIO		5.69 ± 0.62 ^e^

Data are expressed as an equivalent of gallic acid (mg GAE/g) (*n* = 3). Data with different letters in the same column are significantly different (*p* < 0.01).

**Table 3:** Content of soluble phenolic compounds (SPC) in corn varieties.

Sample Code	Kernel Colour	SPC (mg GAE/g) Mean ± SD
OSb	White	1.04 ± 0.13 ^a^
OTb	Yellow	1.60 ± 0.04 ^c^
NSb_2018		1.53 ± 0.07 ^b,c^
NSb_2019		1.24 ± 0.05 ^a,b^
Pop		1.32 ± 0.17 ^a,b,c^
Su/Su		1.35 ± 0.06 ^a,b,c^
Scagliolo		1.23 ± 0.33 ^a,b^
RRb	Red	2.17 ± 0.02 ^d^
PG_2018		1.41 ± 0.02 ^b,c^
PGb_2018		1.52 ± 0.07 ^b,c^
PGb_2019		1.64 ± 0.06 ^c^
Pop BRP	Red/Violet	2.24 ± 0.13 ^d^
Su/Su BRP		4.27 ± 0.43 ^f^
Scagliolo BRP		2.57 ± 0.30 ^e^

Data are expressed as equivalent of gallic acid (mg GAE/g) (*n* = 3). Different letters in the same column correspond to significantly different samples (*p* < 0.01).

**Table 4:** Antioxidant activity (AOA) of rice and corn varieties.

Cereal	Sample Code	Kernel Colour	AOA	
			(mg GAE/g) Mean ± SD	(mg TE/g) Mean ± SD
Rice	OR	White	0.14 ± 0.02 ^a^	1.18 ± 0.10 ^a^
	ERM	Red	0.89 ± 0.03 ^b^	5.28 ± 0.24 ^b^
	KOL		1.70 ± 0.09 ^d^	9.20 ± 0.81 ^c^
	VEN	Black	1.39 ± 0.16 ^c^	8.22 ± 0.69 ^c^
	NRN		2.26 ± 0.09 ^e^	14.25 ± 0.61 ^d^
	VIO		1.65 ± 0.11 ^d^	*
Corn	OSb	White	0.098 ± 0.013 ^a,b^	1.83 ± 0.08 ^a^
	OTb	Yellow	0.134 ± 0.002 ^a,b,c^	2.65 ± 0.12 ^d,e^
	NSb_2018		0.112 ± 0.004 ^a,b,c^	2.61 ± 0.18 ^c,d,e^
	NSb_2019		0.091 ± 0.001 ^a^	2.08 ± 0.06 ^a,b^
	Pop		0.120 ± 0.014 ^a,b,c^	2.15 ± 0.17 ^a,b,c^
	Su/Su		0.197 ± 0.044 ^d^	2.80 ± 0.06 ^e^
	Scagliolo		0.165 ± 0.017 ^c,d^	1.81 ± 0.16 ^a^
	RRb	Red	0.455 ± 0.041 ^f^	3.53 ± 0.12 ^f^
	PG_2018		0.138 ± 0.002 ^a,b,c^	2.19 ± 0.10 ^a,b,c,d^
	PGb_2018		0.151 ± 0.011 ^b,c,d^	2.24 ± 0.15 ^a,b,c,d^
	PGb_2019		0.167 ± 0.010 ^c,d^	2.44 ± 0.11 ^b,c,d,e^
	Pop BRP	Red/Violet	0.354 ± 0.016 ^e^	5.44 ± 0.52 ^h^
	Su/Su BRP		0.736 ± 0.044 ^g^	10.58 ± 0.28 ^i^
	Scagliolo BRP		0.480 ± 0.039 ^f^	4.63 ± 0.66 ^g^

Data are expressed as equivalents of gallic acid (mg GAE/g) for DPPH assay (*n* = 3) and as equivalents of Trolox (mg TE/g) for TEAC assay (*n* = 3). Data with different letters in the same column for the same cereal are significantly different (*p* < 0.01). * Data not available.

**Table 5:** *Ratio frontis* (Rf) and Limit of Detections (LOD) of phenolic acids included in the study.

Compound	*Ratio frontis* Mean ± SD	LOD		
		λ (nm)	ng *	mg/g **
Chlorogenic acid	0.24 ± 0.02	366	100	6.7
Gallic acid	0.45 ± 0.02	254, 366, vis	200	13.3
Dihydroxybenzoic acid	0.54 ± 0.02	vis	40	2.7
Syringic acid	0.58 ± 0.01	vis	100	6.7
Coumaric acid	0.60 ± 0.02	254	200	13.3
Ferulic acid	0.60 ± 0.01	366	100	6.7
Vanillic acid	0.60 ± 0.01	254	100	6.7

* Refers to the amount loaded on to the plate; ** refers to the sample concentration.

**Table 6:** Rice and corn samples included in the study.

Cereal	Sample	Sample Code	Provenience	Pigmentation
**Rice**	Originario brown	OR	Italian market	White
	Ermes brown	ERM	Italian market	Red
	Kolorado brown	KOL	Italian market	Red
	Venere brown	VEN	Italian market	Black
	Nerone brown	NRN	Italian market	Black
	Violet brown	VIO	Azienda Agricola Bertolone	Black
**Corn**	Ostenga brown	OSb	Azienda Agricola Caretto	White
	Ottofile brown 2019	OTb	Azienda Agricola Caretto	Yellow
	Nostrano Isola brown 2018	NSb_2018	Azienda Agricola Caretto	Yellow
	Nostrano Isola brown 2019	NSb_2019	Azienda Agricola Caretto	Yellow
	Rosso di Rovetta brown	RRb	Associazione Rosso Mais	Red
	Pignoletto 2018	PG_2018	Azienda Agricola Caretto	Red
	Pignoletto brown 2018	PGb_2018	Azienda Agricola Caretto	Red
	Pignoletto brown 2019	PGb_2019	Azienda Agricola Caretto	Red
	POP	POP	Bioscience Dept.—UNIMI	Yellow
	su/su	su/su	Bioscience Dept.—UNIMI	Yellow
	Scagliolo	Sca	Bioscience Dept.—UNIMI	Yellow
	POP BRP	POP BRP	Bioscience Dept.—UNIMI	Red/Violet
	su/su BRP	su/su BRP	Bioscience Dept.—UNIMI	Red/Violet
	Scagliolo BRP	Sca BRP	Bioscience Dept.—UNIMI	Red/Violet

## Data Availability

Not applicable.
